# Hydraulic Improvements

**Published:** 1858

**Authors:** 


We copy the following from the *St. Louis Medical and Surgical Journal.* They are said to have been left on the table of Dr. Pope by a patient who evidently had a *tender* appreciation of surgical appliances intended for hydraulic improvements.

"When sorrow's cloud is cast athwart
The sunshine of my mind,
When I, with gloomy care distraught,
No recreation find;
When sighing o'er my hapless lot,
And what I used to be—
I'll seek some quiet, tranquil spot,
And pass a small bougie.
Let strictures on my conduct pass;
Unnoticed let them be:
A stricture somewhere else, alas!
Is more deplored by me.
In hope that blight on manhood's bloom
I yet effaced shall see;
I'll hie me to my quiet room,
And pass a small bougie.
Good bye !"

